# A New Paradigm in the Relationship between Gut Microbiota and Breast Cancer: β-glucuronidase Enzyme Identified as Potential Therapeutic Target

**DOI:** 10.3390/pathogens12091086

**Published:** 2023-08-26

**Authors:** M. Leonor Fernández-Murga, Fernando Gil-Ortiz, Lucía Serrano-García, Antonio Llombart-Cussac

**Affiliations:** 1 Clinical and Molecular Oncology Laboratory, Hospital Arnau de Vilanova-Liria, FISABIO, 46015 Valencia, Spain; luchiserga@gmail.com (L.S.-G.); allombart1@yahoo.com (A.L.-C.); 2 CELLS-ALBA Synchrotron Light Source, 08290 Barcelona, Spain

**Keywords:** breast cancer, microbiota, estrobolome, β-glucuronidase, dysbiosis, inhibitors, personalized medicine, review

## Abstract

Breast cancer (BC) is the most frequently occurring malignancy and the second cancer-specific cause of mortality in women in developed countries. Over 70% of the total number of BCs are hormone receptor-positive (HR+), and elevated levels of circulating estrogen (E) in the blood have been shown to be a major risk factor for the development of HR+ BC. This is attributable to estrogen’s contribution to increased cancer cell proliferation, stimulation of angiogenesis and metastasis, and resistance to therapy. The E metabolism–gut microbiome axis is functional, with subjacent individual variations in the levels of E. It is conceivable that the estrobolome (bacterial genes whose products metabolize E) may contribute to the risk of malignant neoplasms of hormonal origin, including BC, and may serve as a potential biomarker and target. It has been suggested that β-glucuronidase (GUS) enzymes of the intestinal microbiome participate in the strobolome. In addition, it has been proposed that bacterial GUS enzymes from the gastrointestinal tract participate in hormone BC. In this review, we discuss the latest knowledge about the role of the GUS enzyme in the pathogenesis of BC, focusing on (i) the microbiome and E metabolism; (ii) diet, estrobolome, and BC development; (iii) other activities of the bacterial GUS; and (iv) the new molecular targets for BC therapeutic application.

## 1. Introduction

Breast cancer (BC) is the most frequently occurring malignancy and the second most common cancer-specific cause of death in women in developed countries. According to the International Agency for Research on Cancer (IARC) global cancer statistics, 2018 saw more than 2 million new cases and 600 thousand deaths from BC [1]. The estimated 5-year global survival for BC is 98% for localized disease, 84% for regionally disseminated disease, but just 23% for metastatic disease [2].

Lifestyle factors such as Western diet, obesity, alcohol consumption, and a sedentary lifestyle are the principal known risk factors for BC, in addition to other equally relevant factors such as exposure to endogenous and exogenous estrogens (E), high breast density, a history of atypical hyperplasia, and genetic susceptibility [3]. However, less than 10% of BC cases occur due to genetic susceptibility [4]. Hence, the mechanisms of the etiopathogenesis of BC must still be clarified.

The scholarly interest in the role of gut microbiota in human health has exploded since 2010, as reflected in the number of medical publications in prestigious peer-reviewed journals. In recent years, a close relationship between BC and the gastrointestinal tract (GI) microbiome has been suggested. The GI tract hosts over 1000 different bacterial species, and the number of bacteria in humans is estimated to be as high as 10^13^ per gram of luminal content [5,6], which is 10 times the number of human cells in the body. Bacterial load and diversity increase progressively from the stomach to the colon, giving rise to a very complex microbial community [7,8,9]. The composition of the GI tract microbiota (archaea, protozoa, fungi, viruses, and bacteria) reflects host factors, including the mode of delivery, genetics, diet, alcohol consumption, environmental stress, and drugs such as antibiotics and anticancer therapies. 

Advances in 16S ribosomal RNA (rRNA) sequencing and bioinformatics have paved the way for studies in the function and composition of the bacterial microbiome, as well as the assessment of its gene assembly (the metagenome) [10]. Humans and microbes have developed a complex and intricate relationship that benefits the host while allowing the gut microbiota to live in a symbiotic equilibrium. The dysregulation of the microbiome has been correlated with inflammatory, autoimmune, and malignant diseases [11,12,13]. A pathological imbalance within the microbial community may promote oncogenesis, induce tumor progression, and influence responses to cancer therapies and the toxicity profiles of cytotoxic agents when used as antineoplastic agents [14,15,16]. In addition, the human gut microbiome is an active player, exerting effects locally as well as over long distances that include metabolic, hormonal, and immunological messengers [17,18]. Therefore, host–microbe interactions may affect carcinogenesis via various mechanisms including the induction of genotoxic responses, the alteration of the microenvironment, metabolism, and chronic inflammation [19,20].

In this review, we analyze the links between gut microbiota, E metabolism, and BC, and explore the possible implications of β-glucuronidase enzyme substrate metabolites for BC risk, prognosis, and possibly treatment options for more individualized medicine. Finally, we contextualize potential limitations and biases of current microbiota research and suggest ideas for creating novel and solid studies in this exciting and challenging discipline.

## 2. Gut Microbiota, Diversity and Dysbiosis

The gut microbiota consists of microbes, including archaea, protozoa, fungi, viruses, and bacteria that colonize the digestive tract and other areas of the human body. Microbiota is a general term that describes the community of microorganisms that colonize the body, while microbiome refers to the set of genes they encode [21]. 

For the study of the microbiota–microbiome, it is important to define the primary terminology used to understand the differences between a homeostatic microbiota (eubiosis) and an altered microbiota (dysbiosis, a term used to describe a pathological state of gut microbial communities that leads to an intestinal–microbial disequilibrium in the host). Hence, the term α-diversity refers to the abundance of microorganisms in the intestine as assessed by counting operational taxonomic units (i.e., the number of distinct species in the intestine) and the Shannon index (which measures the evenness of the distribution of microorganisms in the intestine). Similar to a microbiological fingerprint, β-diversity is used to compare samples and assess the extent to which the microbial community differs between different environments [7,8,9]. 

The microbiota has the ability to regenerate itself. This quality is known as resilience, which is defined by the capacity to recover equilibrium after an exogenous perturbation (e.g., infection, antibiotic or antitumor treatments such as chemotherapy). These changes can be a pulse disturbance (a limited time interval such as ingestion of a medication), or a press perturbation (continuous stimulus for a prolonged period of time, such as permanent changes in diet or changes in the environment) [7,8,9].

Furthermore, the resilience of the microbiota for dynamic stability is a function of three factors: time (the microbiota composition remains the same over time even in the presence of disturbances), taxonomic groups (group stability across disturbances or over time), and functional groups (even though species and taxonomic clusters may vary, the role of the microbiota remains the same). Only when a level of cumulative stress is reached is there a change from stability into a new equilibrium [7,8,9].

## 3. Microbioma and Estrogen Metabolism: The Estrobolome

**Figure 1:**
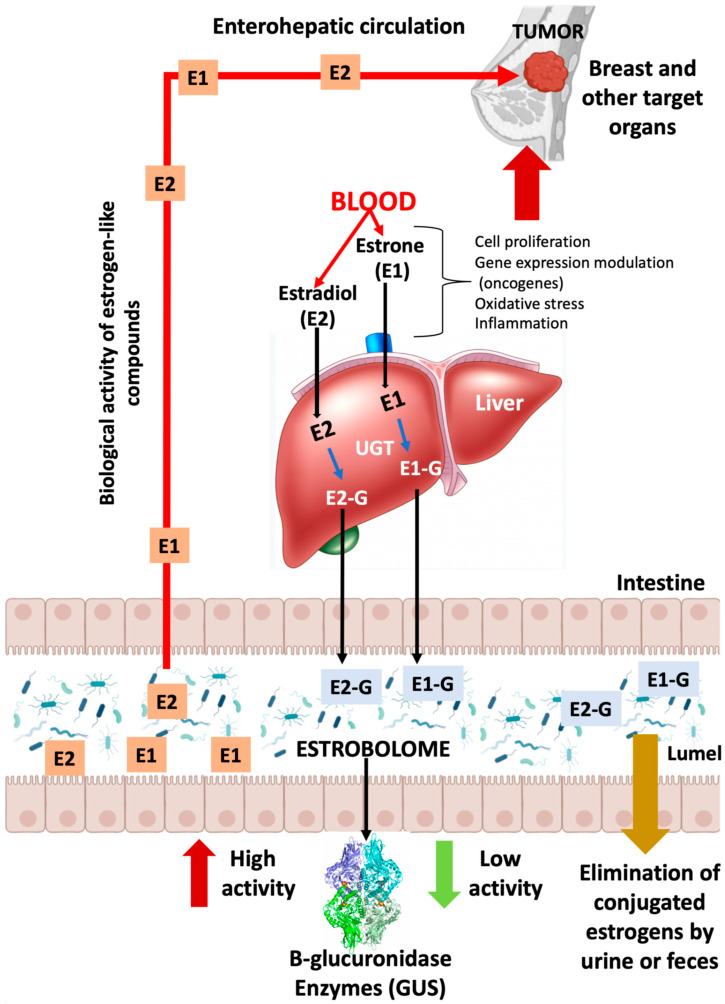
GUS enzymes from the intestinal bacteria that make up the estroboloma release glucuronidated E into the liver via the enzyme UDP-glucuronosyltransferases (UGTs). This reactivation allows E to be recirculated through the portal vein, possibly contributing to hormonal disorders, including breast tumor development. E: estrogen; E-G: glucuronidated estrogen; E1: estrone; E2: estradiol.

An important mechanism of action of the host microbiome is the synthesis of enzymes and the synthesis of its bacterial metabolites. These metabolites can enter the bloodstream, where they are biosynthesized, and then migrate to remote organs, where they develop its biological actions [17]. Bacterial metabolites act as human hormones, and the microbiome acts as an organ that synthesizes them. Then, the blood stream transports bacterial metabolites to the site of action. E metabolism, which involves hydroxylation and conjugation, occurs principally in the liver and involves an enterohepatic circulation pattern. (Figure 1) [17]. Endogenous E exists in three bioactive forms, namely, estradiol (premenopausal), estrone (postmenopausal) and estriol (pregnant women) [22,23]. Conjugated E and metabolites are excreted into the bile and eventually into the GI tract, deconjugating into a variety of E metabolites and, depending on the activity of microbial β-glucuronidases, causing estrogenic activity. (GUS, EC 3.2.1.31) [24]. 

Plottel and Baser refer to the existence of a group of genes present in some enteric bacteria that produce enzymes, such as GUS [22,24], capable of metabolizing E; the sum total of these bacterial genes is known as “the estrobolome” (Figure 1). Therefore, these enzymes are key players in the deconjugation of excreted E, which is important for E reuptake in the distal intestine. Thus, they modulate systemic E availability (via the portal vein) and regulate E-associated pathways. In this scenario, it has been broadly suggested that systemic E and its metabolites (hydroxylated species from estrone or estradiol) can be modulated via the gastrointestinal estrobolome. In human GI, the most important genes coding for GUS are the GUS genes. Mammalian UDP-glucuronosyltransferases bind the glucuronic acid portion to complex compounds, including steroid hormones, labeling them for elimination (more water soluble). Gut microbes possessing GUS genes that encode GUS enzyme activity can remove the glucuronic acid for use as a carbon resource. The resulting aglycones are either secreted into the GI for elimination or reabsorbed into the circulation. Most intestinal bacteria can express GUS enzymatic activity, including *Firmicutes* and *Bacteroidetes* (Table 1). Therefore, these bacterial species affect the levels of E circulating in the blood and excreted in feces and urine. These reactivated E increase their serum levels, which work via the E receptors (ERα and ERβ). The expression of several genes, including mitochondrial genes, is modulated via the activation of these receptors. Increased oxidative phosphorylation has been shown to promote metastasis [25,26], is associated with treatment resistance, and increase tumor aggressiveness [26,27]. In summary, bacterial deconjugation of E favors BC evolution and alters the risk for the occurrence and evolution of E-dependent cancers [26,27,28]. Currently, there is only one pilot study that directly links circulating and excreted E levels in the estrobolome to the presence of BC. Goedert et al. [29], in a pilot study of cases (N = 48) and controls (N = 48) in a population of postmenopausal women, demonstrated that women with BC had a statistically significant modified (β-diversity) composition, and that urine total E positively correlated with α-diversity in healthy women, but not in subjects with BC, signifying lower microbial richness and diversity.

In addition, the enterohepatic circulation metabolizes a number of complex molecules in a similar way, such as neurotransmitters, anticancer drugs, nonsteroidal anti-inflammatory drugs (NSAIDs) and environmental carcinogens. However, it is the intestinal bacteria that largely determine whether they are excreted or reabsorbed into the bloodstream, with the bacterial GUS enzyme playing an active role [30,31] (see Section 5). Consequently, the dysfunction of the E metabolism–gut microbiota axis, in combination with the inherent individual variability in E contents, may promote an elevated incidence of hormone-mediated malignancies, such as BC. In the future, in order to modulate gut bacterial communities with GUS activity and reduce the risk of E-related BC, interventions employing prebiotics, probiotics, postbiotics, or antimicrobials should be considered or used as adjunctive treatments postcancer diagnosis [22,23]. 

**Table 1:** Bacterial species capable of expressing GUS.

Genus	Species	Gene ID ^a^	E Deconjugation ^b^	PDB ^c^ Database (Accession ID)	Reference
*Alistipes*		EXC72_RS02090 ID: 78178623			[25,32]
*Akkermansia*	*muciniphila*	GOZ73_RS09295 ID: 60881251			[31,32]
*Bacteroides*	*Fragilis*	I6J55_RS13335 ID: 66330823	Yes	3CMG	[31,32]
	*cellulosilyticus*	INE78_RS14030 ID: 66307762			[32]
	*intestinalis*	I1224_RS00440 ID: 69505108			[32]
	*uniformis*	INE75_RS18175 ID: 66283800		6NZG, 6D1N, 6D41, 6D50, 6D6W, 6D7F, 6D89, 6D8G	[32,33,34]
	*Ovatus*	Bovatus_RS21525 ID: 29455654		6D8K	[32,34]
	*Dorei*	FYB91_RS01050 ID: 56614211		6ED1	[32,35]
	*massiliensis*	I6J55_RS13335 ID: 66330823			[32]
	*Vulgatus*	GAIMETA21_RS00905 ID: 69838528			[32]
*Bacillus*	*thuringiensis*	A9498_RS29930 ID: 39691567			[32,36]
*Bifidobacterium*	*Dentium*	BIFDEN_RS03045 ID: 69535529		6LD0,6LD6, 6LDB, 6LDC, 6LDD	[32,37]
*Citrobacter*					[32]
*Clostridium*	*perfringens*	uidA [31] ID: 69447906	yes	6CXS, 6JKM,	[32,38,39]
*Collinsella*	*tanakaei YIT 12063*	uidA ID: 62759750			[32]
*Dermabacter*					[32]
*Edwardsiella*	*piscicida*	uidA ID: 72529797			[32]
	*Ictaluri*	uidA ID: 69540280			[32]
*Escherichia*	*Coli*	uidA ID: 946149	yes	6LEG, 3K46, 3K4A, 3K4D, 3LPF, 3LPG, 4JHZ, 5CZK, 6LEG, 6LEJ, 6LEL, 6LEM, 7PR6	[31,32,37,39,40,41,42]
*Eubacterium*	*Eligens*	uidA ID: 41357285	yes	6BJW	[32,43]
*Faecalibacterium*	*prausnitzii*	uidA ID: 56863673 uidA ID: 34751772	yes	6U7I, 6ED2	[32,35]
*Fusicatenibacter*	*saccharivorans*			6NCY, 6NCZ	[32,44]
*Lactobacillus*	*rhamnosus*	RHM_0050 ID: 12473125	yes	6ECA	[32,35]
	*Gasseri*	J3E66_RS04340 ID: 66468975			[32,45]
*Marvinbryantia*					[32]
*Propionibacterium*	*Acnes*	uidA ID: 12534223			[32]
*Parabacteroides*	*Merdae*	DY317_RS05255 ID: 49202940		6DXU	[32]
	*Johnsonii*	HMPREF1077_RS04680 ID: 43351364			[32]
*Roseburia*	*Hominis*	uidA ID: 77458459	yes	6MVH	[32]
	*intestinalis*	uidA ID: 61434358			[32]
*Ruminococcus*	*Gnavus*	N769_RS0107715 ID: 35896210	yes	6EC6	[32,35]
*Streptococcus*	agalactiae	uidA ID: 66885601	yes	4JKL, 4JKK, 4JKL,	[32,39]
	*equisimilis*	GGS_1280 ID: 13799427			[32]
*Tannerella*	*forsythia*	BFO_RS10495 ID: 34759432			[32,39]

^a^ Accession ID are from NCBI. ^b^ Ervin S.M et al. 2019 JBC 294(49): 18586–18599. ^c^ Accession ID are from PDB database.

## 4. Axis Diet, Estrobolome and Breast Cancer

Diet plays an integral role in the complex relationship between human gut microbiota, E metabolism, and their influence on BC recurrence and metastatic potential. The typical Western diet leads to an increased proliferation of undesirable bacteria containing high GUS levels (estrobolome composition). In addition, the bacterial composition of the estrobolome is influenced by host-specific factors, such as age or ethnicity, and by environmental exposures during lifetime, such as alcohol, hormonal treatments, and antibiotic usage. All of these factors impose selective pressure on the bacterial communities of the estrobolome. Although the relationship between the risk of BC and dietary intake has been the subject of intense research, there is still little understanding of the underlying associations or effector mechanisms. Traditionally, high red meat and animal fat intake was linked to an increased BC risk, while high fruit and vegetables consumption was correlated with a decreased risk. These contrasting diets were associated with high and low levels of GUS, respectively [46,47]. For example, the effects of Western diets (high consumption of processed meat, sugars, and fats) appear to be significant in postmenopausal patients with HR+ breast cancer. In contrast, the effects of “healthy” diets, rich in fresh fruit, vegetables, and fish, are only significant in premenopausal women with receptor-positive and receptor-negative tumors [48]. Furthermore, it is well known that the standard Western diet induces diseases such as obesity, insulin resistance, intestinal dysbiosis, and chronic inflammation, all of which are important risk factors for the development of BC [49].

Insulin induces the synthesis of insulin-like growth factor-1 (IGF-1). This protein has been linked to tumor growth and metastasis. In addition, this pancreatic hormone binds to steroid hormone-binding globulin (SHBG), increasing blood E levels and thus contributing to mammary carcinogenesis [50]. In turn, adiponectin levels decrease, leading to insulin resistance and increased IGF-1 levels, inducing cell proliferation. Both E and IGF-1-mediated signaling pathways are increased in obese postmenopausal women. “Cross-interaction” between these pathways represents an important link to tumor progression [50]. Obesity, a condition affecting more than half of postmenopausal women, is a risk factor for BC [51]. The relationship between adult weight gain in women and hormone-dependent cancer risk was confirmed via a meta-analysis of 50 prospective observational studies. This analysis showed that every 5 kg of weight gained is associated with increases in postmenopausal breast (+11%), ovarian (+13%), and endometrial (+39%) cancers [52].

Adipose tissue is known to be metabolically active, with elevated levels of the enzyme aromatase that converts androgens into E, the primary source of E in postmenopausal women. Therefore, excessive E biosynthesis from expanded adipose tissue is associated with adverse disease outcomes in obese women with hormone-sensitive and hormone-resistant cancers [53]. No less important in the relationship between obesity and BC is the chronic inflammatory process from which the obese state originates. This leads to the activation of a large number of metabolic pathways such as JAK2/STAT3, MAPK, EGFR, etc., which are regulators of lipid metabolism, promote chemoresistance, or have mitogenic activity, respectively [54]. However, the molecular and biological basis of obesity in both hormone-positive and hormone-negative breast cancers remains unclear, meaning that more studies are needed on this topic. In addition, there are numerous prominent studies and reviews that clearly associate obesity with gut dysbiosis and its health effects, including BC [55,56,57]. Although not the subject of this review, we should not fail to mention these connections. 

Alcohol consumption is also an important risk factor. High alcohol consumption is associated with disease recurrence and worse rates of survival, primarily in postmenopausal women with E receptor-positive (ER+) BC [58]. Alcohol intake, even less than 10–50 g per day, induces an increased risk of this disease. The European Prospective Investigation into Cancer and Nutrition (EPIC) demonstrated a strong association between alcohol consumption and BC risk in ER+ tumors (N = 360,000 from 10 countries in Europe) [59,60,61,62]. Ethanol intake increases endogenous E levels, especially estradiol and estrone [63]. Ethanol is likely to affect E levels via several different mechanisms: increasing E receptor (ER) expression, activating the steroid hormone signaling pathway, and increasing ER alpha ligand (ER) [63,64,65]. It has been reported in MCF-7 cell models of human BC that ethanol increased ERα expression, aromatase enzyme activity, and cell proliferation. Ethanol has been observed in vitro to stimulate the proliferation of ER+, but not ER-, human CB cells [66]. Furthermore, in hormone-positive MCF-7 and T47D cells, the increase in ERα ligand activity was dose-dependent on ethanol and resulted in the inhibition of BRCA1 tumor suppressor gene expression [67].

Mutlu et al. [68] demonstrated that alcohol consumption can cause small intestinal bacterial overgrowth (SIBO). Both aerobic and anaerobic bacteria are increased in subjects with chronic alcohol intake and alcoholic cirrhosis compared to healthy controls [69]. SIBO and intestinal dysbiosis have also been observed in animal models with alcoholic liver disease [69,70]. However, the interactions between alcohol, E-metabolism, estrobolome, and mammary carcinogenesis in humans still need further attention and definition.

In a recent study by Teng et al., the authors showed that the Mediterranean diet has an inverse relationship, primarily in triple-negative breast cancer patients, favoring an eubiosis (healthy microbiota) [48]. A decrease in GUS activity minimizes circulating E levels and increases SGBH, along with fecal excretion of E. It is known to medicine that intestinal bacteria are involved in this process; however, science is uncertain which bacterial species have high GUS enzyme activity, a pressing issue that we be resolved [19,49,50]. We know that Firmicutes and Bacteroidetes are the intestinal phyla principally responsible for the metabolism of fiber and polyphenols (see Section 5). A diet enriched by vegetable fibers favors the Firmicutes/Bacteroidetes ratio. On the contrary, an intake rich in fats and dairy products increases the species belonging to the phylum Bacteroidetes [71,72]. In addition, the consumption of vegetables and fiber favors beneficial bacteria such as *Prevotella* and *Akkermansia* [71,72]. 

Our gut microbiota, including the estrobolome bacteria, act as potent endocrine regulators, exerting their effects on almost all distal organs by maintaining adequate activity levels [73]. This emphasizes the significance of the relationship between the composition of the microbiota, the biosynthesis of its enzymes, and the production of its metabolites. All these factors must be taken into account if we are to substantially improve cancer research and outcomes [47,48,74,75,76].

## 5. Other Activities of the Bacterial Estrobolome

The bacterial estrobolome also acts on other substrates (androgens, anticarcinogens, polyphenols, phytoestrogens, hetrocyclic amines, etc.) that directly or indirectly negatively or positively affect the development of hormone-dependent BC. 

In the healthy human body, drugs and other xenobiotics are detoxified via glucuronidation in liver (phase II metabolism) by UDP-glucuronosyltransferases (UGTs). This glucuronide molecules are less active, more soluble and excreted by renal clearance and faeces [77]. However, elevated levels of GUS activity revert this process via deglucuronidation and regenerating the active form. In this way, the estrobolome has been implicated in genotoxicity, toxicity, and resistance to therapies [78,79,80]. In this scenario, the term pharmacomicrobiomics arises. This is defined as the science that studies the relationship between the microbiome of the individual and the mechanisms of action and toxicity of drugs [79,80]. 

The discovery of irinotecan (CPT-11), a cytotoxic drug that possesses antiproliferative properties on several types of malignant tumors, including lung cancer, colon cancer, and breast cancer, has revolutionized the applications of camptothecins. Irinotecan is a potent topoisomerase I inhibitor that delays the growth of rapidly proliferating cells in tumors. The potent antitumor activity of irinotecan is due to the rapid formation of an active metabolite in vivo called SN-38. Therefore, irinotecan is a prodrug that generates SN-38 [81,82]. SN38 is detoxified through the addition of glucuronic acid in the liver to form SN38-glucuronic (SN38-G), which is eliminated via the GI tract. The estrobolome removes the glucuronic acid through the GUS enzymes, again releasing the active molecule SN38. Reactive SN38 induces damage to the intestinal epithelium, causing diarrhea and weight loss in the treated patient [82]. Thus, the treatment of toxicities inflicted by irinotecan is a current clinical need. Wallace et al. described several selective inhibitors that block the action of GUS enzymes. In addition, irinotecan induces important changes in the composition of the intestinal microbiota, including an increase in *Proteobacteria* [83,84]. *Proteobacteria*, specifically *Enterobacteriaceae*, have the unique characteristic of possessing a gene operon, encoding GUS enzymes that are hyper-expressed in response to the presence of glucuronidated compounds. This regulatory mechanism allows these bacterial species to utilize glucuronic acid for growth [85]. Also, it has been observed in animal models that, due to their role inhibiting the GUS enzymes of *Enterobacteriaceae* and thereby blocking access to glucuronic acid, GUS inhibitors alone appear capable of curbing the growth of *Proteobacteria* in the gastrointestinal tract. The utility of GUS inhibition also extends to drugs beyond irinotecan and would be relevant in the preventive treatment of postmenopausal women with elevated levels of GUS activity (see Section 7) [38,39,86,87]. 

In contrast, a recent novel study by An. J et al. proposes that the estrobolome can be used in the treatment of hormone-dependent BC [88]. These authors analyzed the composition of the blood microbiome in healthy controls (N = 192 women) and in patients diagnosed with stage 0-III breast cancer (N = 96 women) via NGS (16S ribosomal DNA sequencing). Their pimrary objective was to investigate the GUS and β-galactosidase enzyme-producing estrobolome bacteria involved in E reactivation at the intestinal level. The authors reported that *Pseudomonas* and *Staphylococcus* species were more abundant in healthy controls, while they found that *Enterobacteriaceae*, *Bifidobacterium*, and *Ruminococcaceae* were more frequent in BC patients. GUS-producing bacteria, which were more abundant in diseased subjects, included *Collinsella* and *Edwardsiella*; *Dorea*, *Klebsiella* and *Staphylococcus* were bacteria found to produce β-galactosidase; bacteria that synthesized both enzymes included *Alistipes*, *Bacteroides*, *Bifidobacterium*, *Faecalibacterium*, *Lactobacillus*, and *Roseburia*. Interestingly, *Staphylococcus* was the most frequent species in healthy controls over 40 years of age, and was practically absent in BC patients. In addition, the authors analyzed the effect of extracellular vesicle (EVs) obtained from *S. aureus* cultures on breast cancer cell lines (from MCF7 and BT474 CB) that were treated using tamoxifen. In this simple experiment, they confirmed that the efficacy of tamoxifen increased when administered in combination with bacterial EVs. 

On the basis of these results, the authors suggested that, in the future, a combination therapy of EVs of “good” bacteria, such as *S. aureus*, could be used to improve the efficacy of some breast cancer treatments [88].

A promising therapeutic alternative for the treatment of postmenopausal women is testosterone therapy. Despite its potential benefits, it is necessary to assess the risk of its use in combination with other treatments. Indeed, combined oral estrogen and progestin therapy has been indicated to cause an increased risk of BC [89] due to the high variability of pharmacokinetics and glucuronidation of testosterone after oral administration. Androgen metabolism has been investigated for decades. However, the role of GUS enzymes, produced by intestinal bacteria, in the recirculation and reactivation of testosterone has not been well characterized. In this regard, Basit et al. demonstrated that 5β-dihydrotestosterone (5β-DHTHT) and 3α,5β-tetrahydrotestosterone (3α,5β-THT) are eliminated in feces via hepatic glucuronidation. They also determined the probable recirculation of testosterone glucuronide (THT-G) after its elimination in the intestinal lumen. They showed that the incubation of THT-G with purified intestinal microbial GUS enzymes and human fecal extracts reactivated THT-G by hydrolyzing glucuronic acid to free THT. Therefore, it would be a major breakthrough if were able to learn about the availability and activity of this hormone in patients treated with orally administered testosterone [90].

Consuming red and processed meat has been associated with an increased risk of cancer, which can attributed to exposure to the carcinogenic molecules formed during cooking and conservation processes, such as heterocyclic amines (HCA). Zhang et al. [91] showed that the major form of HCA in the colon is glucuronides (HCA-G), such as *Faecalibacterium prausnitzii*, that have the ability to hydrolyze G-HCA, releasing free HCA. Interestingly, this deglucuronidation reaction, coupled with bacterial glycerol/diol dehydratase activity from *Flavonifractor plautii*, *Blautia obeum* and *Lactobacillus reuteri*, produces metabolites (heterocyclic amines to glycerol conjugates, HCA-M1) with lower mutagenic potential. This study suggests a potential target for modulating estrobolome activities in order to mitigate the risk of HCA carcinogenic activity. 

Polyphenols, which are widely distributed in plants and the human diet, can be found in wine [92], coffee [93], tea [94], fruits, and vegetables [95], are known to have numerous biological activities and can modulate the composition of the gut microbiota, and hence indirectly influence their own metabolism and biovailability [96]. However, humans have a low absorption of these beneficial molecules, and this is largely because their absorption is mediated via coupled metabolic pathways between intestinal bacteria and humans (esterase, glucosidase, demethylation, etc.). The study of polyphenols has become of great interest in the prevention of chronic diseases since epidemiological studies have shown that most of these compounds from the diet have numerous benefits for human health, such as reducing the incidence of cancer [97], cardiovascular disease [98], stroke [99], and type 2 diabetes [100]. 

Finally, polyphenols are the best characterized non-steroidal estrogens. Some intestinal bacteria have been reported to be able to metabolize soybean daidzein isoflavone into equol and/or O-desmethylangolensin (O-DMA), such as *Lactobacillus mucosae*, *Bifidobacterium* spp., *Slackia isoflavoniconvertens*, *Bacteroides ovatus*, *Eggerthella* spp. *strain YY7918*, *Eggerthella* spp. *Julong 732*, *Enterococcus faecium*, *Adlercreutzia equolifaciens*, and *Slackia equolifaciens* [101,102,103,104,105,106,107]. However, only about 30–50% of individuals have intestinal bacteria capable of synthesizing equol. The transformation of isoflavone into equol is directly related to patient diet, the composition and fermentation capacity of the intestinal microbiota, as well as the oxidation–reduction reactions carried out in the intestine [96]. Equol exerts important endocrine effects due to its high binding affinity to the E receptor, primarily activating the estrogen receptor-β (ER-β) [106,107]. As such, it has been used as an alternative treatment in order to reduce menopausal symptoms in women [105]. Anti-androgenic and anti-osteoporotic effects caused by the inhibition of osteoclast maturation have also been reported [108,109]. Investigations in cellular models have revealed antitumor activities by inducing apoptosis and inhibiting cancer cell migration and invasion. Enterolactone, enterodiol, urolithin-A, and 8-prenylnaringenin are synthesized by various intestinal bacteria, such as *Bacteroides* spp., *Clostridium* spp., *Eubacterium limosum*, and *E. lenta*. All of them have the ability to bind to E receptors and inhibit cancer development by inhibiting tumor proliferation and invasion and inactivating angiogenesis [110,111,112].

In the liver, dietary polyphenols are glucuronidated by UGTs enzymes and released into the intestine, where they are hydrolyzed by enzymes of the estrobolome, such as GUS and possibly sulfatases, and returned to circulation via the portal vein [96]. However, this circuit is very complex since it involves a network of enzymes and transporters that make the absorption of these beneficial molecules less effective. The interaction between reactions mediated by hepatic UGTs, intestinal efflux transporters and GUS enzymes present in the intestinal lumen plays a crucial role in recycling (local, enteric and enterohepatic), helping to increase the residence time of polyphenols and their glucuronides in the intestine and liver [113]. It is necessary to clarify in depth the mechanisms of recirculation and the participation of the bacteria of the gut estrobolome in order to develop a detailed understanding of the availability of polyphenols and their link with benefits for human health, primarily in terms of treating BC.

## 6. Gut Microbiota β-glucuronidase Structure 

**Figure 2:**
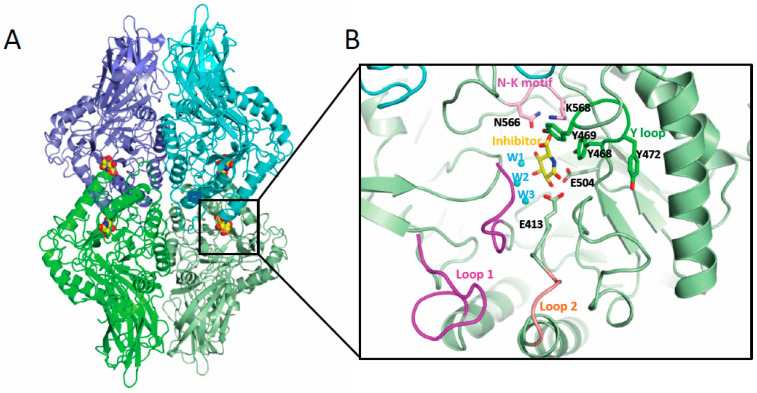
Gut microbial GUS structure. (**A**) Quaternary architecture of GH2 family GUS. The PDB 3K4D corresponding to EcGUS bound to the glucaro-d-lactam inhibitor is shown. Each dimer is represented in different shades of green and blue and the inhibitor is shown in sphere representation (yellow, red and blue represent carbon, oxygen and nitrogen atoms). (**B**) Detail of the active center of one of the subunits showing some structural elements involved in conjugated E binding in GUS enzymes including Loop 1 (magenta), Loop 2 (salmon), N-K motif (pink) and Y loop (dark green). Also, those residues and water molecules (in cyan) involved in substrate binding and catalysis are labelled.

Given the important impact of microbial GUS activity on different forms of cancer, including BC, GUS enzymes have been the target of intensive structural analysis by a significant number of researchers. The first GUS structure corresponding to the human enzyme was established in 1996 [114]. Nevertheless, it was not until nearly 15 years later that the first microbial enzyme structure was characterized as corresponding to the *Escherichia coli* GUS (EcGUS) (Table 1) [40]. To date, more than 40 crystallographic structures of GUS belonging to gut microbiota have been deposited at the Protein Data Bank database (Table 1), along with many others not related with the digestive tract [115]. Microbial GUS enzymes (EC 3.2.1.31) are a broad structural and functional group of enzymes encoded by the uidA gene and included in the glycoside hydrolase families 2 (GH2) classification of the carbohydrate active enzymes (CAZy), possessing exo-β-D-glucuronidase activity. The quaternary structure of bacterial GUS enzymes plays an important role in explaining their biological activity; in fact, with minimal exceptions, the majority of the GUS enzymes show homotetrameric structures (Figure 2A). This is the case with *Bacteroides uniformis* (BuGUS) and *Fusicatenibacter saccharivorans* GUS (FsGUS), which possess dimeric or hexameric architectures [34,44]. Furthermore, the quaternary structure is essential for the deconjugating activity of this enzyme since, in the majority of cases, the N-terminus of the polypeptide chain contributes to the active center formation of the adjacent subunit, playing an important role in the recognition of the aglycone portion of the conjugated E or related substrates [44]. 

In humans, in addition to GUS proteins, another type of enzymes with GUS activity are heparanases (HPSEs), enzymes with endo β-D-glucuronidase activity responsible for the cleavage of heparan sulphate. They constitute a key component of the extracellular matrix, but are not involved in E metabolism [116]. These enzymes, although sharing a similar activity with GUS proteins, are structurally different. Indeed, it is important not to confuse them since HPSEs exhibit a dimeric architecture and belong to the GH79 family [117].

Microbial GUS enzymes are large enzymes with a protomer of around 600 aminoacids, comprising an N-terminal jelly roll β-sandwich domain followed by an immunoglobulin-constant-chain-like domain. The C-terminal sequence includes a core (β/α)_8_ TIM-barrel fold domain containing the glucuronic acid binding site, with the catalytic residues located at the C-terminal end of the central barrel [34,78]. Despite the variability found in the active center of microbial GUS enzymes, there are specific sequence features that are essential for GUS activity over a wide range of glucuronidated substrates, including E glucuronides, as is the case of the key catalytic glutamates, the N-K motif, and the Y loop (Figure 2B). The catalytic glutamate residue, located nearby the loop 2 (E413 in EcGUS) (Figure 2B), interacts with the anomeric hydroxyl group of the glucuronic acid moiety of E-conjugated substrates and promotes proton transfer, acting as a general acid/base, while the second glutamate residue (E504 in EcGUS) (Figure 2B), which interacts with the anomeric and O2 hydroxyl groups, is the residue in charge of the nucleophilic attack [44]. The lysine and asparagine residues belonging to the N-K sequence motif (N566 and K568 in EcGUS) (Figure 2B) recognizes the characteristic carboxylic acid moiety of the glucuronic acid conjugate, representing a crucial signature for differentiating the glucuronic acid moiety relative to galactose [39]. Residues N412 and H330 hydrogen bond the 2-hydroxyl and 3-hydroxyl groups, respectively, while D163 and W549 interact with the 4-hydroxyl group, collaborating to reach the adequate positioning of the glucuronic acid sugar (EcGUS numbering). In addition, the Y loop in GUS enzymes is made up by three tyrosine residues (Y468, Y469 and Y472 in EcGUS) (Figure 2B) near the N-K motif, revealing important structural adaptability and facilitating, through pi-stacking interactions, the binding of E glucuronides [32,39]. Residue Y468 (EcGUS numbering) collaborates in the proper orientation of the nucleophilic glutamate for catalysis, while residue Y472 contributes to the recognition of the carboxylate moiety of the glucuronic acid belonging to an aromatic cage with critical involvement in the binding of E-glucuronides (Figure 2B) [32,34].

The GH2 family includes, in addition to GUS enzymes, β-galacturonidases (GalAses), enzymes capable of cleaving the sugar conjugates of the epimer galacturonate (GalA), instead of glucuronic acid, and also hybrid GUS/GalAses, enzymes with a catalytic machinery capable of differentiating and processing both epimeric substrates [44]. In the case of GalAses, an arginine residue (R337 in *Eisenbergiella tayi* GalAse (EtGalAse) is responsible for the GalA epimer selectivity. In fact, a mutation of this residue abolishes GalAse activity while conferring GUS activity, as occurs in EtGalAse [44]. In hybrid GUS/GalAse enzymes, including BuGUS-1 and FsGUS, the specific arginine residue of GalAse is replaced by a tyrosine (YW motif) that occupies the same position, recognizing the 3-hydroxyl group of both epimers and the axial 4-hydroxyl of GalA [34,44]. In GUS enzymes, the YW motif is occupied by small residues, hence, these sequences are target signatures for differentiating gut microbial GUS. Interestingly, some hybrid GUS/GalAses, such as BuGUS-1, that combine both activities are competent to metabolize E conjugates showing similar catalytic efficiency for either estrone-3-glucuronide or estradiol-17-glucuronide [44].

**Figure 3:**
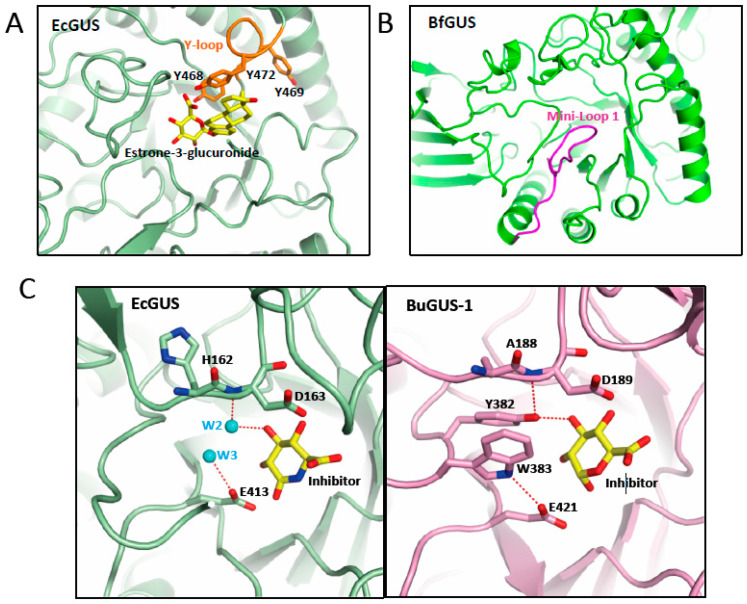
Structural elements involved in the binding of glucuronides. Representation of the active center of GUS enzymes shown in (**A**) EcGUS (PDB 3K4D) (green) with the Y-loop and aromatic cage highlighted in orange and a molecule of estrone-3-glucuronide (yellow) modelled; (**B**) Mini-Loop 1 GUS of *Bacteroides fragilis* (PDB 3CMG) (green) highlighting the location of the mini-Loop 1 (magenta) involved in the binding of E-glucuronides. (**C**) Detail of the interactions of the water molecules 2 and 3 (cyan) in the active center of EcGUS (PDB 3K4D) (green) (left panel) compared with those observed in BuGUS-1 (pink) (right panel) involving residues Y382 and W383.

In addition, the active center of the GUS enzymes is coordinated by a network of water molecules. Seven water molecules were found in the *Bifidobacterium dentium* GUS, interacting with the inhibitor and surrounding residues. Some of these water molecules (W2 and W3) were also found in similar positions in the active center of microbial EcGUS (PDB 3K4D), interacting with the 3-hydroxyl group of the inhibitor and the catalytic residue E413. Hence, they were key players in the binding and deconjugation of E-glucuronides (Figure 2B and Figure 3C) [37]. Interestingly, we have performed active site comparison of these GUS enzymes revealing, that both water molecules are absent in hybrid GUS/GalAses, such as BuGUS1 (PDB 6D6W) (Figure 3C), and their interactions are mimicked by residues W383 and Y382 (BuGUS-1 numbering), belonging to the YW motif, highlighting the relevance of these interactions for the organization of the active center in GUS enzymes. We have observed that the interaction of water molecules (W1) (Figure 2B) with H296 in EcGUS helps to bind the 3-hydroxyl group of both epimers present in either GUS, hybrid GUS/GalAses and GalAses, thereby demonstrating its relevance for substrate binding including E-glucuronides. 

Another key element for the binding of glucuronic acid containing substrates to GUS enzymes is the presence of flexible loops surrounding the active center (Table 2). As a consequence of the wide variability in the length of the amino acid chain of loop 1 and loop 2 (residues 356–380 and 416–419 in EcGUS, respectively), a classification has been created including 7 categories: Loop 1 (bacterial loop) (>15 residues), mini-Loop 1 (10–15 residues), Loop 2 (≥12 residues), mini-Loop 2 (9–11 residues), No Loop and no coverage. The latter category is used in case that sequence information of one of the loops is missing [34]. As a general rule, those enzymes with a center surrounded by longer loops are specialized for the binding and processing of smaller molecule glucuronides, such as p-nitrophenol glucuronide. Conversely, those containing smaller loops, which possess a more open active site, allow for the accommodation and processing of larger substrate-glucuronides, as is the case for a heparosan nonasaccharide substrate [34]. Furthermore, those GUS enzymes specialized in processing smaller substrate-glucuronides, as is the case of the Loop 1 group, tend to have an intracellular location, while the vast majority of them that have the capacity to deconjugate larger substrates are located in the periplasmic space, as indicated by the fact that 78% of GUS enzymes found in human gut microbiota belongs to the Mini-Loop 1, Mini-Loop 2, Mini-Loop 1,2 or No Loop categories [31]. Nevertheless, a few Loop 1 enzymes reveal low affinity towards small substrates, as found in *Ruminococcus gnavus* GUS, is likely due to an alpha helix conformation observed at Loop 1 [35]. The combination of GUS enzymes belonging to different loop categories permits these enzymes to process a wide variability of glucuronic acid-conjugated substrates in the intestinal tract [31]. Those GUS enzymes capable of processing glucuronides of E (estrone-3-glucuronide or estradiol-17-glucuronide) are included mainly in the Loop 1, as is the case of EcGUS or *Clostridium perfringens* GUS (CpGUS), and mini-Loop 1 categories, the latter of which includes *Bacteroides fragilis* and *Roseburia hominis* GUS (Figure 3B) [32]. However, Loop 1 GUS enzymes represent those with the highest catalytic efficiency towards E glucuronides due to their high content in aromatic residues that facilitate its binding, showing a clear preference towards estrone-3-glucuronide. This is likely due to the presence of an extra planar aromatic ring not present in estradiol-17-glucuronide, and also to the different position of the methyl group. Interestingly, GUS enzymes that possess an active site able to accommodate flavin-mononucleotide (FMN) cofactors are also capable of processing glucuronides of E [32]. Since FMN-binding GUS enzymes possess a wider active center, they are able to process both estradiol conjugates with the same efficiency. The steric occlusion caused by a novel 25 residues-long loop nearby the active site in *Faecalibacterium prausnitzii* GUS, a mini-Loop 1 enzyme, appears responsible for its surprisingly low processing activity on E-glucuronide substrates [32].

## 7. Inhibitors of β-glucuronidase as Potential Anti-Cancer Treatment

As mentioned before, gut microbiome GUS enzymes, as active members of the estrobolome, are capable of metabolizing E and other toxic compounds masked with glucuronic acid, as is the case of the colon carcinogen azoxymethane, releasing them into the gut and causing adverse effects that might sometimes be severe, including tumorigenesis [118]. However, blocking these enzymes in the gut by developing specific inhibitors could prevent these adverse effects. An extensive review on GUS enzyme inhibitors was published by Paul Awolade el al. in 2020 [78].

So far, there are a number of selective inhibitors targeting human gut microbiota GUS enzymes which mostly affect GUS activity, even taking into account that druggability studies performed previously reflected the limited predisposition of their active sites to being drug targets [45]. Among them are included some examples of prenylflavonoids (Sanggenon C and Kuwanon G), índole-based compounds (Bazedoxifene), piperazines (Amoxapine) or phenoxy thiophene sulphonamides (BRITE-355252), which show high inhibitory potency in the low micromolar or even low nanomolar range [78]. 

It has been proven that most of the compounds that exhibit high potency towards EcGUS specifically bind to Loop 1 (Inhibitors 2, 3, R1) [40]. Interestingly, some of these compounds (R1, R3, 7, and 8) fail to inhibit other Loop 1 members like the Firmicute enzymes CpGUS and *Streptococcus agalactiae* GUS [39]. The same authors tested the capacity to reduce Irinotecan-induced diarrhea with Inhibitor 1 and R1 in mice, observing an important reduction in symptoms with Inhibitor 1 relative to Inhibitor R1 [119]. Inhibitor 1 was also effective when protecting against the adverse effects caused by nonsteroidal anti-inflammatory drugs [86]. Similar effects were obtained when using other chemotypes. This included pyrazolo[4,3-c]quinolines or amoxapine, which showed comparable efficacy as Inhibitor 1 in the reduction of the side effects of Irinotecan and, in addition, reduced tumor growth in mice [119,120]. Also, probiotic lactic acid bacteria induced beneficial effects by reducing GUS activity in colorectal cancer [118].

**Figure 4:**
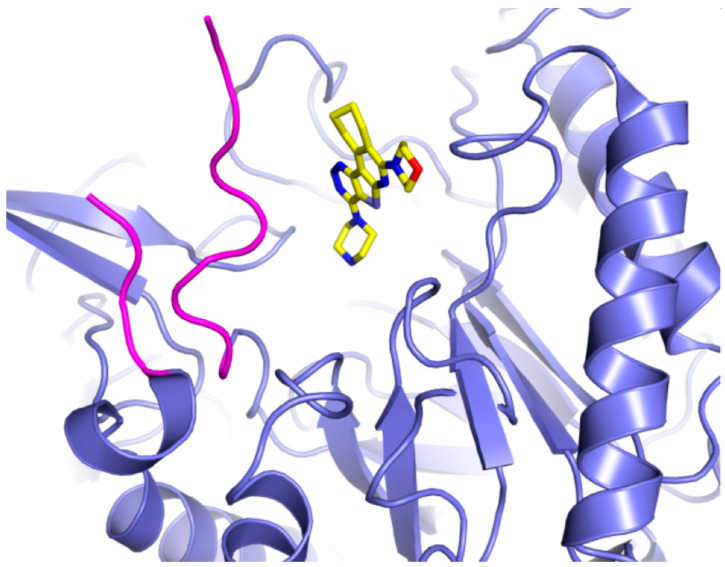
Inhibitor binding to GUS enzymes. UNC10201652 inhibitor represented in sticks is shown bound to the active center of the Loop 1 enzyme CpGUS (PDB 6CXS) (blue ribbon). Loop 1 is colored in magenta.

At the moment, only one inhibitor of gut microbiome GUS enzymes, UNC10201652 (4-(8-(piperazin-1-yl)-1,2,3,4-tetrahydro-[1,2,3]triazino[4′,5′:4,5]thieno[2,3-c]isoquinolin-5-yl)morpholine) or its derivatives, capable of potently inhibiting deconjugation of estrone or estradiol glucuronides, including on fecal samples, has been tested with potency in the low-nM range [32,43]. This compound, which contains a piperazine ring, is a slow-binding inhibitor that targets a catalytic intermediate, showing a preferential effect on those GUS belonging to the Loop 1 group and it has been crystal-bound to CpGUS (PDB 6CXS) (Figure 4) [32,38,43]. Other piperazine-containing compounds have been designed, albeit with less potency [43]. The fact that the enzymes belonging to the Loop 1 group are relatively rare (~5.5% of the GUS enzymes identified in the human intestinal microbiome up to date) [31] explains why the UNC10201652 inhibitor alone is not capable of inhibiting all GUS enzymes belonging to the estrobolome [32]. Nevertheless, a weak inhibition was found in No Loop enzymes (*Bacteroides dorei* GUS), indicating that other interactions, different from those of Loop 1, are involved [35]. It has been hypothesized that this inhibitor could be an effective candidate to prevent tumor growth in an HR+ BC model and, despite the fact that it has been found to be effective in preventing E-glucuronide deconjugation in living *E. coli* cells, it has not been proven effective in transgenic mouse models that exhibit a progression similar to human BC [32]. A study carried out by Bhatt and co-workers compared the in vitro efficacy of the GUS inhibitors UNC10201652 and Inhibitor 1 in terms of reduceing irinotecan-induced gut toxicity, concluding that the first one exhibits higher potency and efficacy towards Loop 1 enzymes as a result of a stronger interaction network in the active site [38]. In this study, the UNC10201652 inhibitor was tested in vivo using a BC mouse model. The authors observed an improvement in tumor regression, a survival increase and a mitigation of the side effects, without significantly affecting the overall metabolism of the host [38,121].

## 8. Future Perspective 

The emergence of massive sequencers that allow up to billions of DNA sequences or fragments (reads) to be read in parallel has revolutionized microbiology, which has moved from an exclusively laboratory setting to a computational one, with the inevitable need for bioinformatics. We can now perform studies of the microbiota, microbiome, and metagenome of a clinical sample quickly and at a fairly affordable cost, allowing us to advance the diagnosis of diseases and the knowledge of the taxonomy and epidemiology of the agents involved. This technique also enables us to carry out comparative genomic studies to discover genes (their variants) and therefore of new metabolic pathways. This can lead to diseases traditionally considered to be of a non-microbial nature being associated with the presence of microorganisms, as in this case with breast cancer. The omics science of metabolites, metabolomics, has also emerged with the incorporation of mass spectrometry and nuclear magnetic resonance into microbiology (NMR). These techniques allow us to accurately and sensitively measure different conjugated and unconjugated estrogens in serum and urine that were previously impossible to identify and differentiate. Currently, all these technological advances complement each other by providing the appropriate tools to make meaningful comparisons between healthy and diseased individuals. Although preclinical animal models and cell cultures are very useful for affirming our hypotheses under controlled conditions, they are not sufficient. We need well-designed observational investigations (controlling for genetic, epigenetic, dietary, and environmental variables) and large-scale investigations in humans in order to identify the associations affecting estrobolome composition and their relationship with BC risk.

Several authors, as discussed throughout this review, argue that the GUS enzymes of the host intestinal estrobolome play a relevant role in the recirculation and reactivation of estrogen. In this scenario, the hypothesis arises that an estrobolome rich in deconjugating GUS enzyme-producing bacteria would be an important risk factor in BC.

The characterization of the structural and enzymological properties of these E-reactivating proteins, the GUS enzymes, in bacteria found in normal individuals and in BC patients could provide us with invaluable information for the modification or modulation of these enzymes. 

GUS enzymes in the intestinal microbiota mostly share the same structural architecture and active center. Although there is wide variability among these enzymes, there are well-defined characteristic sequences that for allow their rapid identification. The processing of a wide variety of substrate sizes in the intestinal tract is made possible by the wide variability of existing GUS enzymes that show important differences in the length of the loops surrounding the active center, with those possessing loop 1 being the most efficient processors of estrogenic glucuronides and, at the same time, the best targets for current inhibitors. Although there is a wide variety of compounds capable of inhibiting the GUS enzyme, so far few of them have demonstrated their efficacy in reducing symptoms and tumor regression, and only one of them has shown high potency in the deconjugation of estrone or estradiol glucuronides. Underexplored active sites should be further inspected for their possible role in the glucuronidation of glucuronidated E. The study of these catalytic sites could help modify GUS enzymes to avoid their E deconjugation potential. These modifications could include alterations to the structure of the active sites involved in deglucuronidation by inducing point mutations in the GUS gene and the deletion of conserved protein motifs, thereby inhibiting their estrogenic reactivation potential. However, genetic manipulation of the human microbiome is not a readily feasible approach. In this scenario, manipulations at the dietary level, such as probiotic or postbiotic supply, are very easy to perform. Therefore, the modification of the estrobolome at the dietary level could be usefully applied to reduce the risk of BC by inhibiting the reactivation of this E-associated protein.

## 9. Conclusions

It is increasingly known, although not sufficiently, that alterations in the metabolism of the estrogen–estrobolome axis associated with individual-specific variations in E levels may contribute to an increased risk of hormone-dependent malignancies, such as breast cancer. Likewise, once BC is established, microbiota could play an important role as prognostic and predictive factors for adequate responses to treatment, the development of resistance to treatment, as well as side effects and toxicities.

Therefore, in the near future, interventions involving the use of gut microbiota modulators such as prebiotics, probiotics, postbiotics, synbiotics, fecal transplantation, and/or antimicrobial agents could be considered. These therapeutic strategies are easy to apply in daily clinical practice and can be designed specifically to allow each subject to have a decreased risk of E-related BC (preventive approach) or, after cancer diagnosis, become adjuvant treatments (personalized therapeutic approach).

## Figures and Tables

**Table 2:** Distinct GUS enzymes architectures and cellular localization.

Phylum (GUS Abundance %)	GUS Loop Classification	Localization	References
*Bacteroidetes* (52%)	L2	Transported across the inner microbial membrane	[31,38,39]
	mL1	Periplasmic space	
	mL2	Transported across the inner microbial membrane	
	NL	Periplasmic space	
	rare mL1,2	Transported across the inner microbial membrane	
*Firmicutes* (41%)	L1	Intracellular	[31,38,39]
	L2	Transported across the inner microbial membrane	
	NL	Periplasmic space	
	mL1	Periplasmic space	
*Verrucomicrobia* (1.5%)	mL2	Transported across the inner microbial membrane	[31,38,39]
*Proteobacteria* (4%)	L1	Intracellular	[31,38,39]

NL: No Loop < 10 residues in Loop 1 region; <9 residues in Loop 2 region. Loop 1 (L1) > 15 residue in Loop 1 region of EcGUS; <9 residues in Loop 2 region. Mini-loop 1 (mL1) contains a loop of 10–15 residues in Loop 1 region; <9 residues in Loop 2 region. Loop 2 (L2) < 10 residue in Loop 1 region; >12 residues in Loop 2 region. Mini-loop 2 (mL2) < 10 residues in Loop 1 region; >9 and <12 residues in Loop 2 region. Mini-loop 1,2 (mL1,2) contain a loop of 10–15 residues in Loop 1 region; >9 and <12 residues in Loop 2 region.

## Data Availability

This manuscript does not include new research data.
